# A Review of Photonic Sintering of Non-Oxide Ceramics for Printed Electronics

**DOI:** 10.3390/ma18102404

**Published:** 2025-05-21

**Authors:** Luis Felipe Gerlein, Mohamad Hassan Taherian, Martin Bolduc

**Affiliations:** Department of Mechanical Engineering, Université du Québec en Trois-Riviéres, Trois-Riviéres, QC G8Z 4M3, Canada; luis.felipe.gerlein.reyes@uqtr.ca (L.F.G.); mohamad.hassan.taherian@uqtr.ca (M.H.T.)

**Keywords:** non-oxide ceramics, photonic sintering, printed electronics, intense pulsed-light sintering

## Abstract

This review article provides a comprehensive analysis of the photonic sintering conditions necessary to process non-oxide ceramics, to obtain similar material properties when compared with those of thermally annealed ones, for various applications in printed electronics. This article presents a thorough examination of the scientific literature on this topic, discussing the principles of photonic sintering applied to non-oxide ceramics, its advantages over traditional post-processing methods, and a quantitative overview of the performance of devices fabricated with the crystalline materials obtained.

## 1. Introduction

Ceramics, in either crystalline or amorphous form, are solid materials composed of metals bonded to nonmetallic elements. These can be generally classified as inorganic, non-metallic materials that can include oxides, carbides, nitrides, borides, silicates, phosphides, sulfides, or their combinations [1,2,3]. Non-oxide ceramics are industry-grade materials that stand out for their high thermal conductivity often exceeding 300 W·$m^{-1}$·$K^{-1}$ at room temperature, making them excellent materials for heat dissipation application electronics, optoelectronics, energy storage, and conversion [4,5,6,7,8]. Additionally, non-oxide ceramics possess exceptional hardness, high melting points, chemical stability, and excellent semiconducting properties, opening a plethora of applications in mechanical tools, aerospace, hydrophilic coatings, semiconductors, and optoelectronics and as part of temperature-resistant structural parts [4,5,9,10,11,12,13,14]. Finally, non-oxide ceramics exhibit a wide range of electronic bandgaps that determine their role in electronic applications. For example, TiC and WC are narrow-bandgap materials (<1 eV) and are used for electrodes and heating elements, while SiC and BN are wide-bandgap materials (>3 eV), and their applications lie in insulators, optoelectronics, and UV photonics [15,16,17].

It is these electronic properties that make some of the well-known non-oxide ceramics valuable in the evolving field of printed electronics. Among carbides, Silicon Carbide (SiC), Tungsten Carbide (WC), Boron Carbide (B_4_C), and Titanium Carbide (TiC) are sought after for their thermal stability, specially in high power applications [5,6,8,18]. In the case of nitrides, Silicon Nitride (Si_3_N_4_), Boron Nitride (BN), Aluminum Nitride (AlN), and Titanium Nitride (TiN) stand out for their already-extensive list of electronic uses that can be extended into printed applications [19,20,21,22,23,24,25]. Borides, such as Titanium Diboride (TiB_2_), Zirconium Diboride (ZrB_2_), Hafnium Boride (HfB), and Lanthanum Hexaboride (LaB_6_), offer excellent stability in highly demanding environments as thermal protection systems (TPSs), expanding the potential applications of printed electronics [26,27,28]. Common sulfides include Molybdenum Disulfide MoS_2_, Tungsten Disulfide (WS_2_), Tin Sulfide (SnS), Copper Sulfide (Cu_2_S, Cu_x_S), and Zinc Sulfide (ZnS). These show good electrical and optical properties for printed light-emitting devices, photodetectors, photovoltaics, and TFTs [9,29,30,31,32].

Due to their inorganic nature, chalcogenides and perovskites are often represented as a specialized subset with distinct properties within the non-oxide ceramics. Nonetheless, there is ongoing research pursuing the fabrication of printed devices using deposition techniques such as inkjet, aerosol, screen, blade, or transfer printing [33,34,35,36]. In a similar fashion, abundant research is available on the photonic post-processing of these materials [37,38,39,40,41,42,43,44].

### 1.1. Post-Processing of Non-Oxide Ceramics

Due to their ceramic nature, traditional post-processing methods for these materials often involve high-temperature, energy-intensive, time-consuming, and oftentimes atmosphere-specific sintering techniques to achieve a high degree crystallinity, excellent material densification, and those desirable electrical and mechanical properties [11,45].

Alternatively, modern additive manufacturing (AM) deposition and sintering techniques have found their way into the processing of non-oxide ceramics. Recently, fused filament fabrication (or fused deposition modeling, FDM) for material extrusion, selective laser sintering (SLS), stereolithography, and vat photopolimerization (VP) have been demonstrated as routes of manufacturing for specific applications [25,46,47,48,49,50,51,52]. However, more research is required to yield fully dense and low internal stress materials, high-quality surfaces, accurate geometries, and acceptable interlayer adhesion [47,53,54].

### 1.2. Photonic Sintering Fundamentals

**Figure 1:**
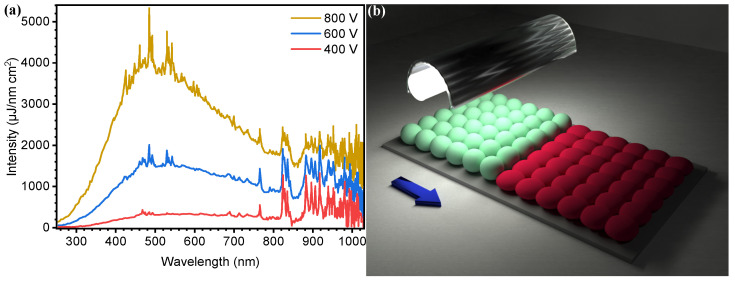
(**a**) Xenon lamp emission spectrum. Data obtained from NovaCentrix (Austin, TX, USA), provider of PulseForge^®^ photonic curing tools, and (**b**) PS schematic representation. The arrow symbolizes the direction of movement of the sample and the transformation of particles to a sintered film represented from green to red spheres.

Photonic sintering (PS), also known as intense pulsed-light (IPL) sintering or flash lamp annealing (FLA), is a post-processing method used in AM of printed electronics to sinter a variety of inks, from nanoparticle-based to polymeric and molecular for applications in printed circuits, solar cells, gas sensing, and photocatalysis, among others [40,55,56,57,58,59,60]. This technique has also been explored for surface modification to improve material adhesion properties [61,62]. It provides advantages in reduced waste, rapid prototyping, and the possibility of process high-temperature materials on a variety heat-sensitive, flexible, and transparent substrates [57,63,64]. This technique employs high-energy, short-duration pulses of broadband light emission from a xenon lamp covering from 380 to 950 nm, shown in Figure 1a. These light pulses achieve rapid heating at the surface of thin films of materials, where subsequent sintering, soldering, crystallization, or modulation of chemical reactions takes place, thanks to the rapid propagation of the generated heat throughout the film, as illustrated in Figure 1b [65,66,67].

#### Photonic Sintering of Ceramic Materials

Indeed, post-processing of ceramics using PS is a valid alternative over traditional thermal-driven sintering processes because of the substantial acceleration of the sintering kinetics, rapid prototyping, and overall reduction in production costs [58,68]. However, it is important to highlight the lower equipment cost of thermal solutions when compared with photonic sintering equipment. Additionally, thermal sintering is highly tolerant to small variation in processing parameters because it is a thermally equilibrated process where, for instance, a small variation of 10 °C on a 1000 °C sintering process will not have a great impact on the final product. In contrast, the multiplicity of parameters that can be controlled on a photonic sintering process such as pulse duration, lamp voltage, processing distance, micro-pulse shaping, repetition rate, and the presence of other materials, added to its non-equilibrated heating nature forces a careful processing design [69]. As such, the PS of ceramics is limited to the particular cases where thin films are used in the fabrication of printed electronic devices. There is, however, substantial research published around the PS conditions necessary to process a wide variety of printed metal oxides due to their high electronic mobility, transparency, and lower temperature processing [58,68,70,71,72,73].

### 1.3. Scope of This Review

In contrast with the availability of research for the PS of oxide ceramics, research focused on the PS of non-oxide ceramics is sparse and incipient at this time. This review aims to gather the most relevant up-to-date publications that explore the PS of non-oxide ceramics for applications in printed electronics. Indeed, non-oxide ceramics offer unique properties, and their ability to maintain structural integrity under extreme conditions makes them attractive for advanced printed electronics applications.

## 2. Photonic Sintering of Non-Oxide Perovskites

Out of the all the non-oxide ceramics, photonic sintering non-oxide perovskites and its effects in device operation is the most extensively studied in the literature. Non-oxide perovskites are a subclass of perovskite materials in which the anion, the X in ABX_3_, is not oxygen. Instead, it is typically a halide (e.g., fluoride, chloride), nitride, hydride, or a combination of such elements. Reports of non-oxide Perovskites treated by PS sintering include MAPbI_3_, MAPbI_3−x_Cl_x_, MAPbBr_3_, and MAPbBr_3−x_I_x_ perovskites, all used for the fabrication of photovoltaic devices [42,43,44,74,75,76]. While very sensitive to temperatures exceeding 150 °C, where the perovskite layer degrades into PbI_2_ and MAI, reported photovoltaic devices where the active layer was photonically sintered performed close to their thermally annealed counterparts. This takes into account that only during a few milliseconds the perovskite layer can substantially exceed the theoretical limit of 150 °C, thanks to the careful control of other photonic sintering parameters [41,42].

### 2.1. Influence of PS in the Perovskite Film Morphology and Crystallinity

**Figure 2:**
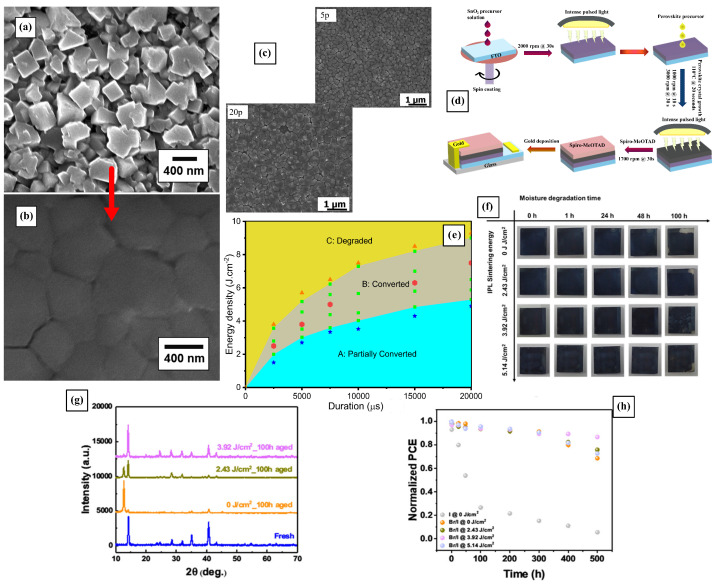
(**a**) SEM images of perovskite films as deposited and (**b**) films processed with PS with a 2000 J, 2 ms pulse [42]. The red arrow indicates the film’s before and after states. (**c**) SEM images of PVP-added perovskite films treated with 5 and 20 pulses of 26.5 J·${\mathrm{cm}}^{-2}$ and 2 ms duration (**d**). Example of multiple PS steps used to sinter different constituents on a PSC [74] (**e**). Plot showing how multiple PS optimized parameters yield complete crystallization of the perovskite films. The mapping shows the partially converted turquoise zone with the star data points; the fully converted in the gray zone with the squares and hexagons data points, and the degraded yellow zone with the triangle data points [75]. Perovskite films processed with PS show much better moisture stability in humid environments, illustrated in the pictures of moisture aging experiments in (**f**), the XRD patterns in (**g**), and the evolution of PCE of the PSC device over time in (**h**) [76].

The grain size, crystallization speed, and coverage can be strongly affected by the PS parameters such as energy density and pulse duration [40,41]. It has been demonstrated that higher energy pulses increase the crystallite size and sinter of the films, yielding larger perovskite grain sizes that reduce phase separation in mixed halide perovskite films [41,42,75,76]. Lavery et al. initially showed that, at a pulse energy of 2000 J, perovskite crystals increase in size and sinter together to form a dense layer, shown in Figure 2a,b [42]. Later, Ankireddy et al. showed that the surface coverage and grain growth were improved by using polyvinylpyrrolidone (PVP) as a surfactant during the photonic sintering process [43]. Indeed, the PS of films without PVP can result in phase-segregated MAPbCl_3_ and MAPbI_3_ perovskites, while PVP-added films exhibit suppressed MAPbCl_3_ formation, resulting in layers of fused and recrystallized mixed halide perovskites (MAPbBr_3−x_I_x_). As a consequence, these PS perovskites showed improved solar cell performance represented in better charge transfer, optimized bandgap, and improved conversion efficiency [43,76]. For samples containing PVP, an increase in the number of 26.5 J·${\mathrm{cm}}^{-2}$ pulses from 5 to 20 resulted in a reduction in the XRD peak intensity at 15.7°, corresponding to MAPbCl_3_. SEM images of the films treated with either 5 or 20 pulses are presented in Figure 2c. In contrast, the same increase in pulse number led to an opposite trend in films that did not contain PVP [43]. MAPbI_3_ films absorb pulsed light, reaching temperatures exceeding 250 °C and recrystallizing into larger grains, and the PS treatment can help realign the crystal orientation [42,76].

Other intermediate dopants or surface treatments acting in synergy with the PS processing also yield improved film morphology and grain size. Ghahremani et al. showed that the addition of diiodomethane (CH_2_I_2_) improved surface coverage, grain growth, and film quality by increasing the solution boiling point and delaying the unfavorable natural crystallization of the as-spinning solution. These improvements were observed when firing five pulses of 2 ms pulse duration at an irradiated energy of 1.4 kJ [74]. In a similar fashion, the enhancement of the perovskite morphology by increasing the grain size and reducing the defects was successfully achieved by Slimani et al. by using chlorobenzene’s (CB) ability to create an intermediate phase that darkens the film, increasing light absorption and perovskite crystallization [75]. Indeed, CB-treated films were more compact and uniform and exhibited large grains averaging 851 nm, with excellent crystallization shown by the XRD analysis, all thanks to the careful optimization of the pulse parameters, cleverly depicted in the diagram in Figure 2e. Indeed, an array of processing parameters are suitable to obtain crystalline perovskites; however, careful analysis of the device’s performance and other metrics showed light on the best set of processing conditions. The optimal PS parameters were 3.52 J·${\mathrm{cm}}^{-2}$ with a pulse duration of 5000 µs, which resulted in better performant devices compared with thermally annealed ones.

### 2.2. Influence of PS in the Optical Properties of the Perovskite Film

Higher light absorption is observed in PS-processed samples compared with thermally annealed films as a result of the better surface coverage and crystallinity [43]. The absorption band edge for perovskite films was to be determined around 450 nm, which translated into a bandgap of approximately 2.9 eV, indicating mixed-halide perovskite formation. However, PVP-added films processed with PS can show decreased absorption potentially due to PVP decomposition during the photonic sintering process [43].

Photoluminescence analysis of PS films showed that the PL peaks were sensitive to both energy density (ED) and pulse duration [75]. The highest intensity peak with a narrow full width at half maximum (FWHM) of 21 nm was observed with the optimal PS processing parameters of 3.5 J·${\mathrm{cm}}^{-2}$ for 5000 µs, indicating a reduction in trap states and non-radiative recombination. Interestingly, a redshift in peak wavelength occurred at a lower pulse energy density (2.5 J·${\mathrm{cm}}^{-2}$ to 5 J·${\mathrm{cm}}^{-2}$), followed by a blueshift beyond the optimal values, related to grain size distribution and phase transition in MAPI.

### 2.3. Influence of PS in the Electrical Properties and Overall Device Performance

Troughton et al. demonstrated that crystallized CH_3_NH_3_PbI_3−x_Cl_x_ films using 1.15 ms pulses with 3.99 J·${\mathrm{cm}}^{-2}$ of energy density show slower charge recombination than thermally annealed films, which helps maintain a high open-circuit voltage ($V_{OC}$) [40]. However, their devices showed a lower PCE of 11.3% for the PS samples versus 15.2% obtained through 90 min thermal annealing. The difference in PCE was attributed to the smaller perovskite crystals obtained through PS and the absence of capping layers in the film’s surface. This work emphasizes the significance of parameter optimization in PS for achieving high-quality perovskite films.

The benefits of photonic sintering are not limited to the crystallization of the perovskite layer in PSCs, showing that sequential steps of PS enable rapid device fabrication, as illustrated in Figure 2d. This was shown by Ghahremani et al., where they explored the effects of PS in the SnO_2_ electron transport layer using 5 pulses at 2.1 kJ and the triple-cation perovskite layer using 5 pulses at 1.4 kJ, demonstrating a reduction in the photoluminescence (PL) intensity of the SnO_2_ layer, suggesting less charge recombination at the SnO_2_/perovskite interface and improved charge transport [74]. The authors also explored the performance difference between rigid glass substrates and flexible PET ones, showing that flexible PSCs had lower performance compared with rigid cells, attributed to the higher sheet resistance of ITO coated sheets, handling during fabrication, and the unsuitability of spin coating for forming uniform films on smooth ITO coated sheets.

While devices with and without PVP fabricated using PS exhibited photovoltage, interestingly, the devices without PVP showed higher performance parameters across the board (Voc, Jsc, FF, and PCE) despite the inferior surface coverage against the PVP–added ones. This is because of the higher-than-normal bandgap obtained for the MAPBI_3_ layer for the PVP–added devices, confirmed by UV–VIS absorbance and XRD results [43].

### 2.4. Influence of PS in the Perovskite Film Stability

PS-sintered perovskite films showed improved moisture stability, maintaining their morphology and photoelectric conversion efficiency (PCE) under high humidity conditions, portrayed in Figure 2f–h. As demonstrated by Peng et al., perovskite films sintered at 3.92 J·${\mathrm{cm}}^{-2}$ showed almost no signal of PbI_2_ after 100 h in a 70% relative humidity environment [76]. PS enhances crystallinity and reduces defects in the perovskite film, while substantially reducing the fabrication time and costs. These factors are crucial for improving the overall efficiency and stability of solar cells [75].

## 3. Photonic Sintering of Chalcogenides

Photonic sintering has gained attention as an alternative to traditional high-temperature treatments for chalcogenide materials used in solar cells and thermoelectric devices [33,38]. Indeed, compared with traditional thermal treatments, which often require high temperatures, e.g., >400 °C for Bi_2_Te_2.7_Se_0.3_, and long processing times, limiting the choice of substrates and increasing manufacturing costs, photonic sintering offers several advantages for processing chalcogenides. PS can achieve comparable or even superior results in seconds, minimizing thermal damage to the substrate and enabling the use of flexible or temperature-sensitive materials.

### 3.1. Influence of PS in the Morphology and Crystallinity of Chalcogenide Films

**Figure 3:**
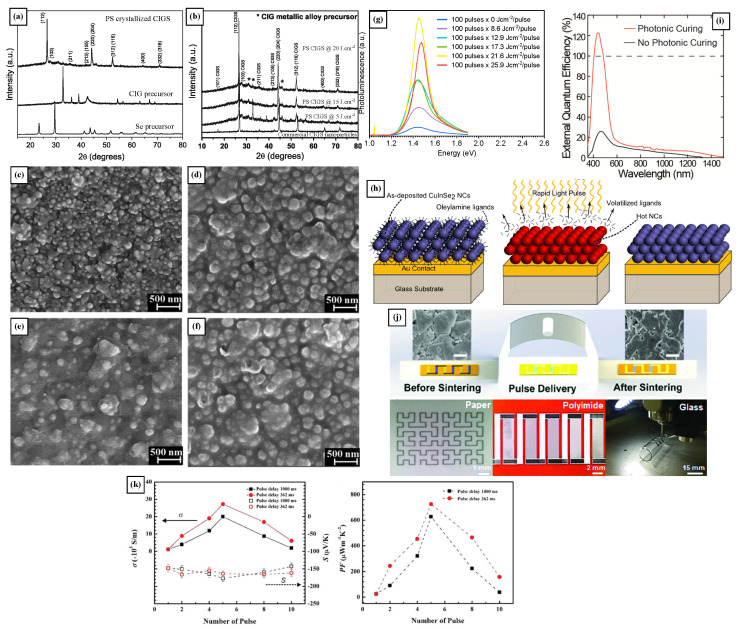
(**a**) XRD signals of the Se and CIG precursors versus that of the fully crystalline CIGS film by PS [37] (**b**) XRD pattern of commercially available CIGS nanoparticles and CIGS films processed at various EDs [37]. The topological variations of CdTe films PS-treated with different numbers of 21.6 J·${\mathrm{cm}}^{-2}$ pulses: (**c**) 80, (**d**) 90, (**e**) 100, and (**f**) 110 [38] (**g**). Photoluminescence of PS-treated samples varying the pulse fluence [38]. (**h**) Use of PS to remove oleylamine capping ligands from the CuInSe_2_ nanocrystal film creating quantum dot solids, while maintaining the nanocrystal size and the quantum confinement effects [39]. (**i**) EQE spectra from solar cells made with CuInSe_2_ both as deposited and processed by PS at 2.2 J·${\mathrm{cm}}^{-2}$ [39]. (**j**,**k**) Fabrication of the PS-treated TE devices made of Bi_2_Te_2.7_Se_0.3_ showing the before and after PS, printing in various substrates and the comparison of Seebeck coefficients and power factor of devices processed with pulses of varying duty cycles [33].

In a work presented by Dhage et al., non-vacuum PS in environmental conditions was applied on a metallic alloy of Cu(In_0.7_Ga_0.3_) (CIG) and Se nanoparticles to form copper indium gallium diselenide (CIGS) films, without the need for toxic selenization or high-temperature vacuum conditions, which are neither cost-effective nor easily scalable to high-volume production [37]. The tetragonal chalcopyrite structure was confirmed by XRD, and the grain size in the these CIGS films, processed at an ED of 20 J·${\mathrm{cm}}^{-2}$, ranged from 0.3 µm to 1 µm, for films 4 µm thick, shown in Figure 3a,b. An energy of at least 5 J·${\mathrm{cm}}^{-2}$ was needed to initiate CIGS formation but left some CIG unreacted in the films, while using 20 J·${\mathrm{cm}}^{-2}$ resulted in almost complete conversion of the precursors, as shown in the XRD patterns presented in Figure 3a,b, where the signals from each precursor are compared with that of the final CIGS crystalline phase and that of the commercial CIGS nanoparticles. The film processed using PS showed compact grains separated by grain boundaries with very few voids, while avoiding the oxidation of the elements and second-phase generation [37]. The elemental composition of the 4 µm CIGS films processed at 20 J·${\mathrm{cm}}^{-2}$ maintained stable stoichiometric proportions of Cu:In:Ga:Se = 25.04:15.99:7.67:51.3, when compared with the precursors’ initial concentrations, Cu:In:Ga:Se = 25:16:7.5:50, which resulted in single-phase films. However, the low photoactivity of the material was attributed to a remnant of the CIG precursor in the films [37].

Cadmium Telluride (CdTe) thin films also undergo significant transformations upon PS treatment, as can be appreciated in the SEM micrographs from Figure 3c–f. Dharmadasa et al. found that 100 pulses of 21.6 J·${\mathrm{cm}}^{-2}$ were optimal for recrystallization and defect reduction in electrochemically deposited CdTe films [38]. Scanning electron microscopy (SEM) revealed a unique characteristic of the films processed by PS: the formation of a continuous melted layer on the surface, effectively eliminating pinholes and voids, creating a high-quality thin film with a preference towards the (111) crystalline plane, shown in Figure 3c–f. Lower energy densities resulted in smoother particle surfaces, while higher energy densities led to the formation of larger grains (up to 1 µm) and increased surface roughness. This report showed how the appropriate selection of the pulse’s ED influences the intensity of the (111) XRD peak when other parameters such as the number of pulses were left unchanged. The crystallinity was maximized at an ED of 21.6 J·${\mathrm{cm}}^{-2}$ while higher ED resulted in the degradation of the CdTe film, lowering the XRD peak intensity, and suggesting a loss of material or structural degradation [38].

Saeidi-Javash et al. reported the use aerosol jet printing and photonic sintering to fabricate flexible thermoelectric films from Bi_2_Te_2.7_Se_0.3_ nanoplates, shown in the images from Figure 3j [33]. The optimal PS pulse parameters were found to be 5.1 kW·${\mathrm{cm}}^{-2}$ in pulse power density, 5 ms pulse duration, and 5 repetitions, with a pulse delay of 362 ms in between pulses. These parameters were systematically found balancing power density, pulse duration, and pulse repetition by assessing the processing–structure–property correlations in the films. Initially, the printed nanoplatelets of Bi_2_Te_2.7_Se_0.3_ were isolated by the PVP surfactant, which was then removed by the PS processing resulting in coalesced nanoplatelets. Higher power densities resulted in large pores and over-sintered films. The optimal parameters led to the highest grain size, porosity, electrical conductivity, carrier mobility, and specially power factor, when compared with other flexible n-type thermoelectric materials. The thermoelectric performance of TE devices fabricated using PS of varying duty cycles is shown in the plots of Figure 3k [33].

Through careful control of the pulsed-light parameters, photonic sintering can also be used for the evaporation of solvents or ligands in different applications, before further reactions such as sintering of crystallization take place [77,78]. In a work reported by Stolle et al., the main objective was to enhance charge transport in nanocrystalline CuInSe_2_ films by using PS while avoiding high-temperature selenization. To this end, the oleylamine ligands surrounding the CuInSe_2_ nanocrystals were removed without inducing significant grain growth, which improved substantially the electrical contact between the nanocrystals, graphically depicted in the schematic of Figure 3h [39]. However, the authors noted that PS can introduce traps coming from unpassivated surface defects in CuInSe_2_ films, which reduce PV device performance under low-light conditions. After the PS treatment using a 160 µs, 2.2 J·${\mathrm{cm}}^{-2}$ single pulse exposure, Jsc increased substantially from 5.65 to 18.65 mA/${\mathrm{cm}}^{2}$, while Voc decreased from 0.41 to 0.21 V, and the comparison of EQE is shown in Figure 3i.

### 3.2. Influence of PS in the Optical Properties of Chalcogenide Films

In the CdTe films reported by Dharmadasa et al., the optical bandgap only changed from 1.47 eV on the as-deposited films to 1.46 eV on those treated with the optimal PS conditions, alongside optical transmittance of over 50% on the red portion of the spectrum. Additionally, there was an increase in intensity and sharpening of the PL peak, as can be appreciated in Figure 3g. These improvements arose from reducing the density of donor and acceptor traps that acted as defects in the material. PS treatment with energy densities higher than 21.6 J·${\mathrm{cm}}^{-2}$ resulted in a intensity reduction and blueshift of PL peak, indicating deteriorated optical and electrical properties in the films [38].

In the CuInSe_2_ films reported by Stolle et al., there was a redshift of ∼70 meV in the bleach peak position in the transient absorption (TA) spectrum after curing, suggesting a change in the electronic structure of the material, which is consistent with a slight loss of quantum confinement resulting from the loss of oleylamine capping ligands. At the optimal PS conditions, the average size of the nanocrystals went from 8.1 nm to 9.2 after sintering at 2.2 J·${\mathrm{cm}}^{-2}$ and to 23.1 nm after sintering at 2.5 J·${\mathrm{cm}}^{-2}$, showing how a small variation in the PS parameters will have strong influence in the resulting properties of the films [39].

### 3.3. Influence of PS in the Electrical Properties and Device Performance of Chalcogenide Films

Films of CdTe crystallized using PS where subject to photoelectrochemical (PEC) measurements by immersion in an aqueous solution of 0.1 M of Na_2_S, as a way to quantify its effect in the crystallization of the films. The improvements in grain size, crystallinity, and defect density resulted in better photocurrent generated [38].

Similarly, the electrical conductivity of Bi_2_Te_2.7_Se_0.3_ printed films increased with increasing the PS pulse duration up to a certain point after which; this effect was reversed for longer pulses. Additionally, multiple pulses yielded better electrical performance than a single pulse exposure, going from non-conductive to $2.7\times 10^{4}$ S·$m^{-1}$, and their carrier mobility (µ) went from 8.2 to 25 ${\mathrm{cm}}^{2}$·$V^{-1}$·$s^{-1}$ at five pulses, and then dropped by almost 50% at eight pulses using the optimal PS parameters [33]. These films were used as thermoelectric (TE) sensors showing a power factor of 730 µW·$m^{-1}$·$K^{-2}$ at room temperature, substantially surpassing any other flexible n-type TE materials reported up-to-date.

## 4. Photonic Sintering of Other Non-Oxide Ceramics

Photonic sintering is primarily applied to thin films due to the intense heat generated by the absorption of short light pulses, which is concentrated at the surface and rapidly dissipates through the film [66]. Beyond non-oxide perovskites and chalcogenides, research on the photonic sintering of non-oxide ceramics remains limited. This is largely due to the stringent thermal conditions required for crystallization and the challenges associated with fabricating films of sufficient thickness [68].

To date, only the work of Wirth et al. has demonstrated the application of PS in the post-processing of Al-doped SiC [79]. The recrystallization of p-type doped 6H-SiC using photonic sintering after aluminum ion-implantation was achieved using 20 ms long pulses, reporting very high Al doping (⩾$5\times 10^{20}{\mathrm{cm}}^{-3}$) and low resistivity (0.01 Ω·cm). These long pulses generated surface heat of about 2000 °C, facilitating the SiC crystal healing and preventing diffusion of the implanted aluminum ions.

**Figure 4:**
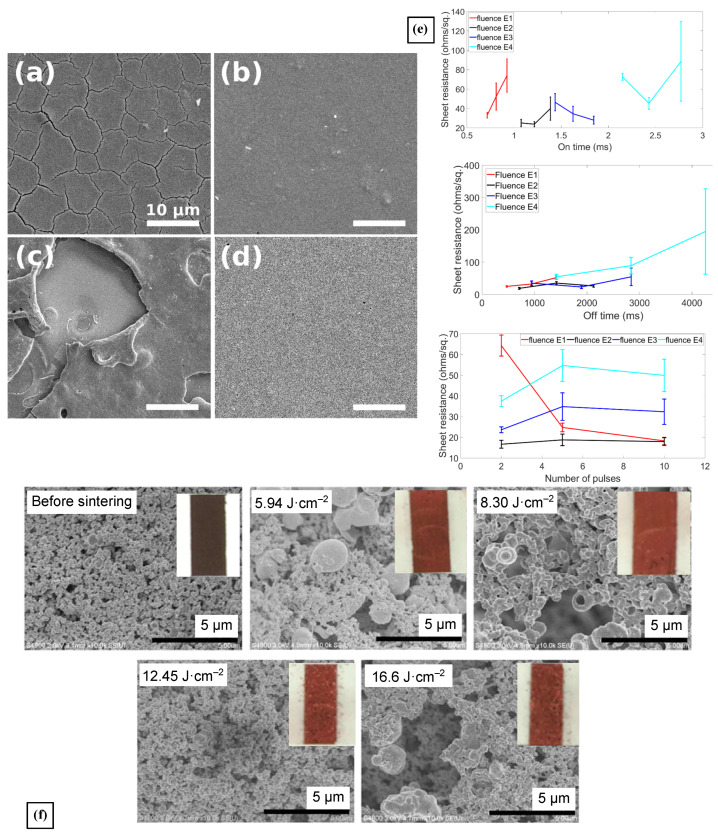
(**a**–**d**) SEM images of CZTS nanocrystal coatings processed with PS on Mo-coated SLG (**a**,**c**) and Mo foil (**b**,**d**). (**a**,**b**) were processed with a single 9 J·${\mathrm{cm}}^{-2}$ pulse, while (**c**,**d**) with 10 pulses of 11.6 J·${\mathrm{cm}}^{-2}$ each. The blistering of the CZTS film is most notorious at high PS energy density [80]. (**e**) These three plots show how physical structure of the Cu_x_S films reacts to the variation of one parameter of the PS process while leaving the others fixed: top image is on-time; middle image is off-time; and bottom image is the number of pulses [81] (**f**) SEM images of films prepared using vehicle 2 after PS processing with different irradiation conditions. The inset photos show the final Cu film originated from the Cu_3_N ink [82].

Williams et al. investigated the assembly of thin films that started from Copper Zinc Tin Sulfide (Cu_2_ZnSnS_4_, CZTS) nanocrystals by means of PS for applications in solar cells [80]. The authors explored a variety of pulse conditions for processing: pulse fluence from 3.9 to 11.6 J·${\mathrm{cm}}^{-2}$ and pulse repetition number from 1–400. The pulse repetition rate and the pulse duration were fixed at 4.3 Hz and 3.5 ms, respectively. All the chosen pulse parameters were tested in three different substrates aiming to determine the influence of their thermal expansion in the appearance of cracks and blisters in the CZTS thin films. It was found that both a high number of pulses and high pulse ED (10 pulses and ⩾9 J·${\mathrm{cm}}^{-2}$, respectively) resulted in blistering, elemental decomposition, and ablation of the CZTS films, as shown in Figure 4a–d. Additionally, the blistering was the result of the interaction between the CZTS nanocrystals and the Mo-coated substrate, creating vapor pockets. Additionally, the authors correlated the repeated thermal expansion and contraction of the thin films with the appearance of high-density cracking (Figure 4a) [80]. These are well-known effects of photonic sintering when the films have solvents that need evaporation before the material annealing takes place. The authors demonstrated that switching substrates from a low to a high thermal diffusivity substrate helps mitigate the presence of high-density cracks.

The effects of PS in copper sulfide (Cu_x_S, x = 1 to 2) thin films were studied for the first time by Bansal et al. The film’s dominant crystalline phase, covellite or digenite, was determined by the post-processing conditions, and thus, its possible applications ranging from absorbers to solar cells and thin-film electronics [81]. Indeed, at a fixed number (5) of pulses, different concentrations of the digenite (Cu_1.8_S)–covellite (CuS) mixed phases were obtained at lower pulse fluence values (5 and 7.5 J·${\mathrm{cm}}^{-2}$), and at higher fluence values (10 and 15 J·${\mathrm{cm}}^{-2}$), there was a phase transformation from a mixed covellite–digenite to a pure digenite phase, as a result of the increased film temperature. A similar behavior was measured when increasing the ON/OFF ratio of the pulse (duty cycle) as longer ON times also result in elevated heating of the film. Conversely, longer OFF times meant more cooling of the film in between pulses, which limited its heating, and thus, more control over the mixed phases. Interestingly, a higher number of pulse repetitions leaving every other condition fixed yielded higher concentrations of covellite over digenite. As expected, higher digenite/covellite ratios meant higher sheet resistance in the films. In contrast, retention of the covellite phase while increasing the grain growth resulted in better electrical performance, which was then correlated to the variation of the PS parameters to find the optimal processing conditions, whose impacts in the sheet resistance of the film is shown in Figure 4e. A reduction of nearly 99% in the $R_{sh}$ of the films was observed when processed at 7.5 J·${\mathrm{cm}}^{-2}$, 3 pulses, and ON/OFF times of 1.215/761 ms (0.159% duty cycle), when compared with the as-deposited film with $R_{sh}$ = 3.66 kΩ·${\mathrm{sq}}^{-1}$ [81]. This work strongly highlights the versatility of PS showing a flexibility that is non-existent with traditional oven sintering methods.

Dexter et al. elaborate on the results obtained in [56,81] by applying a similar analysis to the study and model of the phase transition dynamics that nanoparticles Cu_x_S undergo under PS processing [83]. The authors demonstrated that the loss of sulfur is the driving factor that yields the covellite-rich film first, which then degenerates into the digenite-rich film, as a result of the intense heating generated by the PS process [81,83].

Nakamura et al. explored the effects of PS in the fabrication of printed copper wiring, starting from a copper nitride (Cu_3_N) ink [82]. Cu_3_N is an advantageous material for flexible printed circuits because it thermally decomposes into metallic copper at much lower temperatures than CuO and Cu_2_O, 188 °C vs 500 °C and 333 °C, respectively. It exhibits the highest light absorbance properties of the three copper compounds, making it an excellent candidate to be processed by photonic sintering on temperature-sensitive substrates. With the inclusion of ethylene glycol in the formulated liquid ink, the authors managed 99% of the Cu_3_N into metallic copper, using both PS conditions of 12.45 and 16.60 J·${\mathrm{cm}}^{-2}$, 1 pulse, and 1000/500 and 1000/2000 µs ON/OFF ratios. At the highest fluence, the Rsh of the Cu film was 0.7 Ω·${\mathrm{sq}}^{-1}$; however, the mechanical integrity of the film suggested poor copper particle necking after PS. It was necessary to use chemical additives to minimize the intensity of the copper reaction during PS. With these, the adhesion, particle necking, and mechanical properties of the paste inks were substantially improved, albeit a reduction in the copper conversion to 80%. Figure 4f shows the impact of varying the ED in the processing of the Cu_3_N ink using vehicle 2. In the end, the optimal conditions to process the paste inks were found to be 4 pulses of 8.3 J·${\mathrm{cm}}^{-2}$ and 2000 µs pulse duration with 50% duty cycle to obtain a $R_{sh}$ of 0.506 Ω·${\mathrm{sq}}^{-1}$, demonstrating the potential of Cu_3_N for wiring inks in printed electronics applications [82].

**Figure 5:**
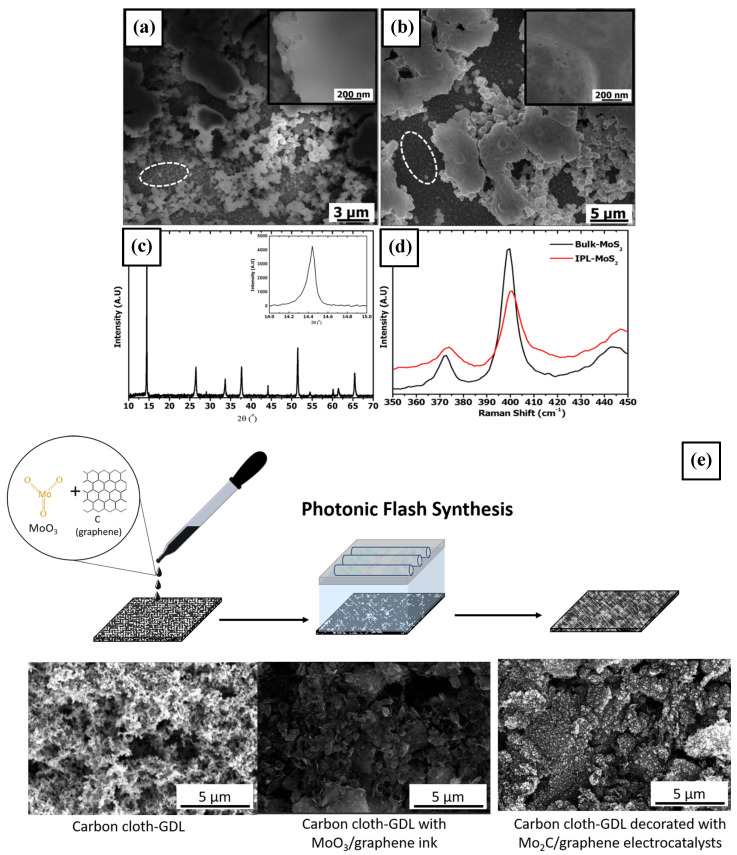
SEM images showing the structure of the MoS_2_ films as deposited in (**a**) and after photonic sintering in (**b**). The areas surrounded by white dashed ellipses are magnified in the insets of (**a,b**). XRD and Raman characterization of the photonic sintered MoS_2_ film in (**c**,**d**) © IOP Publishing. Reproduced with permission. All rights reserved [84]. (**e**) Schematic showing the two-step protocol to fabricate the Mo_2_C/graphene electrocatalyst, showing the effects of using photonic sintering [85].

The formation of thin films of defect-rich MoS_2_ from a precursor of ammonium tetrathiomolybdate using PS was demonstrated for their use in water splitting applications through hydrogen evolution reaction (HER) in a work published by Gupta et al. [84]. The reported PS process parameters are pulses of 25 J·${\mathrm{cm}}^{-2}$, with a 2 ms pulse duration, and the number of pulses is 10, demonstrating the how the presence of defects in the produced MoS_2_ thin films is beneficial to the HER reaction. The topological images shown in Figure 5a,b highlight the appearance of these defects after PS, while the characterization in Figure 5c,d confirms the formation of the MoS_2_ crystalline phase when compared with the bulk material. Indeed, molybdenum-based electrocatalysts are an attractive alternative to replace noble metals used in HER reactions for the production of hydrogen. Another electrocatalyst produced using PS is molybdenum carbide (Mo_2_C) presented in a report by Reynard et al. [85]. Here, the decomposition of molybdenum oxides and graphene ink into βMo_2_C when placed on a carbon cloth substrate, using 4 pulses from the xenon lamp, yielded highly crystalline material with small traces of oxides and high activity in the acidic conditions of the HER experiments. The SEM images presented in Figure 5e show each of the steps, from the carbon cloth to the mixed MoO_3_+graphene ink and the final Mo_2_C structure embedded in the substrate. The reported PS conditions involved 4 pulses with a pulse fluence of 12.8 J·${\mathrm{cm}}^{-2}$ with a duration of 20,000 µs and a pulse shaping of 18 µ-pulses evenly distributed throughout each pulse.

## 5. Conclusions

Table 1 summarizes the essential parameters utilized in the PS of materials, including pulse duration, energy density, pulse repetition rate, ON/OFF times, or duty cycle, when available. These parameters play a critical role in determining the resulting material structure, morphology, and properties. It is important to note that, because of the non-equilibrated nature of this processing technique, the parameters reported are not absolute, and it is possible to obtain similar results with the combination of parameters [75,81,82,83,86].

Photonic sintering (PS), also commonly known as intense pulsed-light (IPL) sintering, describes the post-processing of thin films with high energy and short flashes of unpolarized white light emanated from a xenon lamp. This leads to expeditious, high-throughput, cost-saving, high-temperature thermal processing and ultra-rapid densification of a variety of materials atop a similarly wide variety of substrates, from glass to heat-sensitive flexible polymers—a critical requirement for flexible electronics. These short pulses of light, in the range of tenths of microseconds to milliseconds, can be tailored to deliver specific amounts of energy density by varying the charging voltage, the ON/OFF times, and the number of pulses to carefully influence the material’s response to the pulsed light. This flexibility allows for drying, sintering, annealing, crystallizing, polymerizing, and modulating chemical reactions, hence the variety of names for such technique. This review has provided the reader with a clear overview of how PS has been applied to a wide variety of non-oxide ceramics, focusing on the effects on the materials’ morphology, crystallinity, optical and electrical properties, and device performance where available.

Despite these advantages, photonic sintering of non-oxide ceramics presents inherent limitations that necessitate further investigation. A primary challenge resides in achieving homogeneous densification, particularly over large-area films, because the depth of light penetration is highly material dependent. This heterogeneity becomes more pronounced in non-oxide ceramics, which often exhibit strong optical absorption and higher refractive indices, leading to reduced light penetration depth and, consequently, diminished cured thicknesses, often less than 50 μm [52]. Such shallow curing depths can result in weak interlayer bonding and an increased susceptibility to defects such as cracking and porosity during subsequent processing steps [52].

Furthermore, the localized and rapid temperature increase characteristic of photonic sintering can induce significant thermal gradients within the material. In ceramics prone to strong optical absorption, this may lead to localized overheating, potentially causing cracking, incomplete sintering, or uncontrolled phase transformations. The inherent brittleness of many non-oxide ceramics exacerbates this issue, as thermally induced stresses can readily exceed the material’s fracture toughness [87]. Indeed, observations in specific material systems have shown that elevated pulse energy densities or numerous pulses can result in blistering, elemental decomposition, or ablation of the non-oxide ceramic films. Blistering, for instance, has been associated with the rapid vaporization of solvents or interactions between the film and the underlying substrate [80].

The efficacy of the photonic sintering process is also directly contingent on the optical absorption characteristics of the target material at the emission wavelengths of the light source. Certain non-oxide ceramics may exhibit suboptimal absorption within the typical broadband emission spectrum of xenon lamps, thereby reducing the efficiency of energy transfer and hindering effective sintering. Moreover, while photonic sintering can rapidly achieve high temperatures, the potential for oxidation or other atmospheric reactions at the material’s surface, especially for ceramics sensitive to ambient conditions, remains a consideration, even with short processing durations [88]. Finally, the seamless and effective integration of photonic sintering equipment and methodologies with existing additive manufacturing workflows and established microfabrication techniques continues to be an area of active research and development. Addressing these limitations is important for the broader application of photonic sintering in the processing of non-oxide ceramic materials.

Undeterred by these challenges, PS is poised to redefine the post-processing of non-oxide ceramics, as demonstrated in this review. The capability to rapidly process nanostructured ceramic powders and thin films opens new avenues for engineering ceramics with tailored optoelectronic, thermoelectric, and electrochemical properties. PS is particularly instrumental in enabling the development of non-oxide ceramics with engineered microstructures, enhanced charge transport properties, and tunable surface functionalities.

Looking forward, the potential of non-oxide ceramics in flexible and printed electronics is substantial. These materials offer distinct advantages over polymeric and metallic counterparts, particularly in applications demanding high thermal stability, superior electrical performance, and chemical resilience. PS serves as a pivotal enabler in this transition, providing a high-throughput, scalable approach that aligns with the evolving demands of printed electronics. Future research should prioritize optimizing light-matter interactions in non-oxide ceramics to enhance sintering uniformity, developing wavelength-specific sintering protocols tailored to distinct ceramic compositions, and integrating PS with emerging additive manufacturing platforms to enable the fabrication of complex high-performance ceramic architectures.

## Figures and Tables

**Table 1:** Summary of the processing parameters used in the main references explored in this review.

Material	Pulse Fluence (J·${\mathrm{cm}}^{-2}$)	Pulse Duration (ms)	Duty Cycle	Repetitions	Reference
MAPbBr_3−x_I_x_ + PVP	26.5	2 ms	N/A	5–20	[43]
Cs_0.05_(MA_0.85_ FA_0.15_)_0.95_ PbI_3_ + CH_2_I_2_	1.4 kJ ^(1)^	2	N/A	5	[74]
MAPbI_3_ + CB	3.52	5	50%	1	[75]
CH_3_NH_3_PbI_3−x_Cl_x_	3.99	1.15	N/A	1	[40]
MAPbBr_3_ MAPbBr_3−x_I_x_ MAPbI_3_	3.92	1	N/A	1	[76]
Cu(In_0.7_Ga_0.3_)Se_2_	20	2	N/A	1	[37]
CdTe	21.6	1	N/A	100	[38]
Bi_2_Te_2.7_Se_0.3_	27.1	5	1.4%	5	[33]
CuInSe_2_	2.2	160	N/A	1	[39]
Al-doped SiC	N/A	20	N/A	N/A	[79]
Cu_2_ZnSnS_4_	3.9–11.6	3.5	N/A	1–400	[80]
Cu_x_S	7.5	762.215	0.159%	3	[81]
Cu_3_N	16.60	2	50%	4	[82]
MoS_2_	12.8	20	18 µ-pulses	4	[84]

^(1)^ In this source, only the power delivered was provided. N/A: relevant information not available in the cited source.

## Data Availability

No new data were created or analyzed in this study.

## Abbreviations

The following abbreviations are used in this manuscript:

PS	photonic sintering
IPL	intense pulsed-light
PSC	perovskite solar cell
ED	energy density
PV	photovoltaic
TE	thermoelectric
Voc	open-circuit voltage
Jsc	short-circuit current
FF	fill factor
PCE	power conversion efficiency
PVP	polyvinylpyrrolidone
CB	chlorobenzene
HER	hydrogen evolution reaction
